# Analgesic Activity, Chemical Profiling and Computational Study on *Chrysopogon aciculatus*

**DOI:** 10.3389/fphar.2018.01164

**Published:** 2018-10-15

**Authors:** S. M. Neamul Kabir Zihad, Niloy Bhowmick, Shaikh Jamal Uddin, Nazifa Sifat, Md. Shamim Rahman, Razina Rouf, Muhammad Torequl Islam, Shrabanti Dev, Hazrina Hazni, Shahin Aziz, Eunüs S. Ali, Asish K. Das, Jamil A. Shilpi, Lutfun Nahar, Satyajit D. Sarker

**Affiliations:** ^1^Pharmacy Discipline, Life Science School, Khulna University, Khulna, Bangladesh; ^2^Biotechnology and Genetic Engineering Discipline, Life Science School, Khulna University, Khulna, Bangladesh; ^3^Department of Pharmacy, Faculty of Life Science, Bangabandhu Sheikh Mujibur Rahman Science and Technology University, Gopalganj, Bangladesh; ^4^Department for Management of Science and Technology Development, Ton Duc Thang University, Ho Chi Minh City, Vietnam; ^5^Faculty of Pharmacy, Ton Duc Thang University, Ho Chi Minh City, Vietnam; ^6^Centre for Natural Products and Drug Discovery, University of Malaya, Kuala Lumpur, Malaysia; ^7^Chemical Research Division, Bangladesh Council of Scientific and Industrial Research, Dhaka, Bangladesh; ^8^Department of Biochemistry and Molecular Genetics, Northwestern University Feinberg School of Medicine, Chicago, IL, United States; ^9^Medicinal Chemistry and Natural Products Research Group, School of Pharmacy and Biomolecular Sciences, Liverpool John Moores University, Liverpool, United Kingdom

**Keywords:** *Chrysopogon aciculatus*, poaceae, analgesic, hot plate test, liquid chromatography mass spectrometry, gas chromatography mass spectrometry, aciculatin, docking

## Abstract

Present study was undertaken to evaluate the analgesic activity of the ethanol extract of *Chrysopogon aciculatus*. In addition to bioassays in mice, chemical profiling was done by LC-MS and GC-MS to identify phytochemicals, which were further docked on the catalytic site of COX-2 enzymes with a view to suggest the possible role of such phytoconstituents in the observed analgesic activity. Analgesic activity of *C. aciculatus* was evaluated by acetic acid induced writhing reflex method and hot plate technique. Phytochemical profiling was conducted using liquid chromatography mass spectrometry (LC-MS) and gas chromatography mass spectrometry (GC-MS). In docking studies, homology model of human COX-2 enzyme was prepared using Easy Modeler 4.0 and the identified phytoconstituents were docked using Autodock Vina. Preliminary acute toxicity test of the ethanol extract of *C. aciculatus* showed no sign of mortality at the highest dose of 4,000 mg/kg. The whole plant extract significantly (*p* < 0.05) inhibited acetic acid induced writhing in mice at the doses of 500 and 750 mg/kg. The extract delayed the response time in hot plate test in a dose dependent manner. LC-MS analysis of the plant extract revealed the presence of aciculatin, nudaphantin and 5α,8α-epidioxyergosta-6,22-diene-3β-ol. Three compounds namely citronellylisobutyrate; 2,4-dihydroxy-7-methoxy-(2H)-1,4-benzoxazin-3(4H)-one and nudaphantin were identified in the *n*-hexane fraction by GC-MS. Among these compounds, six were found to be interacting with the binding site for arachidonic acid in COX-2 enzyme. Present study strongly supports the traditional use of *C. aciculatus* in the management of pain. In conclusion, compounds (tricin, campesterol, gamma oryzanol, and citronellyl isobutyrate) showing promising binding affinity in docking studies, along with previously known anti-inflammatory compound aciculatin can be held responsible for the observed activity.

## Introduction

Bangladesh, with its subtropical weather and fertile deltaic land, is rich in a variety of medicinal plants. The traditional medicine system of Bangladesh has developed over generations through trial and error. Bangladesh is the habitat of more than 500 medicinal plant species and the rural people have long depended on them for their primary healthcare needs (Ghani, 2003). Currently, about 250 medicinal plants are being used for the preparation of herbal formulations in Bangladesh and the estimated market value of medicinal plants is around 3.3 billion BDT (4.1 million USD) (Ghani, 2003; Bachar, 2013). The traditional healers of Bangladesh utilize these medicines of plant origin to treat various disease conditions including inflammation, hypertension, asthma, diabetes, gastro-intestinal disorders, cardiac problems, and skin diseases among different localities and communities throughout the country (Ghani, 2003; Rahmatullah et al., 2010a,b; Kadir et al., 2012; Islam et al., 2014).

*Chrysopogon aciculatus* (Retz.) Trin. of the Poaceae family is a vigorous creeping grass commonly growing in Bangladesh. Locally in Bangladesh, it is known as Premkata, Chorkata, Chui-kanta, and distributed all over the country. This tropical grassland species also grows in many other Asian countries including Malaysia, India, Nepal, Philippines, China and Indonesia, where it is commonly known as ‘Love Grass’(Ghani, 1998). A study conducted in Nepal revealed that this grass species is a popular cattle feed constituting 45% of total grazed pasture in lowland areas (Lehmkuhl, 1992). It is an exotic grass in Nigeria, where it is well established as a lawn grass (Stanfield, 1970). This medicinal herb plays an important ethnomedicinal role in different regions across the world and extensively used for the management of wide range of ailments. In lieu of diverse traditional uses, a few medicinal property of this weed has been evaluated and reported so far. In Bangladesh, the Garo ethnic community from Madhupur, Tangail uses *C. aciculatus* root juice to cure liver pain and the whole plant of *C. aciculatus* to treat cattle leg swelling, where it is known by the name Negraban and Negra bam (Anisuzzaman et al., 2007). Investigations revealed *C. aciculatus* to be rich in biologically important phytoconstituents belonging to several chemical classes including glycosylflavones, flavonols, sterols, flavones and germacranolides (**Supplementary Table S1**).

As a part of our continuing research on medicinal plants of Bangladesh, present study was undertaken to evaluate the local ethnomedicinal use, the antinociceptive activity of the ethanolic extract of *C. aciculatus* on animal model and to identify bioactive phytoconstituents by chemical profiling through LC-MS and GC-MS (Mondal et al., 2014; Sheikh et al., 2016; Zihad et al., 2018). Analgesic activity was observed in both peripheral and central model of analgesia. Furthermore, computational study was conducted with the identified compounds against human COX-2 enzyme. The aim of this study was to draw a scientific basis to the traditional use of this plant in mitigating pain among the traditional healthcare practitioners of Bangladesh.

## Materials and Methods

### Materials

The plant *Chrysopogon aciculatus* (Retz.) Trin. (Family: Poaceae) was collected from Khulna University area, Gallamari, Khulna-9100, Bangladesh on April, 2014. The plant material was identified by the experts of Bangladesh National Herbarium, Bangladesh where a voucher specimen (DACB 45183) has been submitted for future reference. Reference drugs, Diclofenac sodium and Morphine, were generously provided by Beximco Pharmaceutical Ltd., Bangladesh and Popular Phamaceutical Ltd., Bangladesh, respectively.

### Extraction

Collected plant parts were separated from undesirable materials and were washed with water. Sun-dried plant material was ground to coarse powder with the help of a suitable grinder (Capacitor start motor, Wuhu motor factory, China). The powdered material was soaked in ethanol for 5 days with occasional shaking and stirring. The crude extract was obtained through filtration and evaporation of the solvent with the aid of a rotary evaporator.

### Experimental Animals

Young Swiss Albino mice, aging 4–5 weeks and weighing 25–28 g, were purchased from Animal Resources Branch of International Centre for Diarrhoeal Disease Research, Bangladesh (ICCDR,B). The animals were acclimatized with an ambient temperature of 25 ± 2 °C; 12 h dark-light cycle and 56–60 % relative humidity under pathogen free condition. Our study was approved by the Animal Ethics Committee, Pharmacy Discipline, Life Science School, Khulna University, Khulna-9208, Bangladesh (Protocol Number: KU/PHARM/AEC/15/006/025).

### Identification of Phytochemical Constituents

Presence of different classes of phytochemicals in the ethanol extract of *C. aciculatus* including reducing sugar, tannins, flavonoids, saponins, gums, steroids, alkaloids, glycosides, and terpenoids were investigated by standard procedures.

### Acute Toxicity Test

Test mice were randomly divided into control and test groups containing six mice of either sex in each group. Test groups were administered with graded doses (62.5–4000 mg/kg b.w., p.o.) of *C. aciculatus* extract while control group received vehicle (1% Tween 80 in water, p.o.). Then the animals were observed for 72 h for mortality and any sign of toxicity (Lorke, 1983).

### Acetic Acid Induced Writhing Test

Animals of either sex were divided into negative control, positive control and test groups. Thirty min prior to the administration of acetic acid (0.7%, 10 ml/kg b.w., i.p.), control and positive control group were treated with vehicle (1% Tween 80 in water, p.o.) and standard diclofenac sodium (25 mg/kg b.w., p.o.) respectively, while the test groups received *C. aciculatus* extract at the doses of 500 and 750 mg/kg b.w. orally. After 5 min of administration of acetic acid the animals were observed for 10 min and the number of writhes by each group was recorded (Sheikh et al., 2016).

### Hot Plate Test

Experimental animals were treated with control vehicle (1% Tween 80 in water, p.o.), morphine (5 mg/kg b.w., i.p.) and *C. aciculatus* extract (500 and 750 mg/kg b.w., p.o.). They were then placed onto a hot plate maintained at 55 ± 0.5°C to induce pain stimulus on each 30 min starting from the treatment throughout the observation period of 2 h. Response time i.e., time taken for paw licking or jumping was recorded as a measure of the analgesic effect of the treatment. A cut off time of 15 min was maintained to prevent any possible injury to the experimental animals (Sheikh et al., 2016).

### LC-MS Analysis

LC-MS analysis was conducted using Agilent 6530 Accurate-Mass Q-TOF LC-MS system equipped with a C18 analytical column of 50 mm × 2.1 mm, 1.8 μm particle size (Agilent 6530). Column oven temperature of 35°C and flow rate of 250 μL/min were maintained throughout the experiment. Water and acetonitrile, each containing 5 mM ammonium formate and 0.1% formic acid, were used as mobile phase A and B, respectively. The injection volume was 20 μL with a run time of 15 min. The linear gradient program was set as follows: 0 min, 100% A; 0–45 min, 0–100% B; 46–50 min, 100% B; 51–55 min, 100% A. The UHPLC was hyphenated to a triple quadrupole mass spectrometer 3200 QTrap (ABSciex) equipped with an electrospray ionization interface set at negative mode. The interface heater held at the temperature of 500°C and an ion-spray (IS) voltage of -4500 eV. The nebulizing gas (GS1), heating gas (GS2) and curtain gas pressures set at 40, 40, and 10 psi, respectively during the whole analysis. Nitrogen was used as collision and spray gas. Full scan data acquisition was performed, scanning from *m/z* 5 to 1500 in enhanced MS IDA EPI mode (Karim et al., 2014).

### GC-MS Analysis

GC–MS analysis was performed using an Agilent 6890 N Network GC System equipped with an Agilent 7683B Series auto-injector, coupled to an Agilent 5975 Inert Mass Selective Detector. The operating conditions were as follows: initial oven temperature, 50°C for 5 min, then to 150°C at 4°C/min and held for 5 min, then to 250°C at 4°C/min and held for 10 min; injector and detector temperatures, 275^o^C; injection volume, 0.2 μL; split ratio, 50:1. The carrier gas used was He at 1.0 mL/min. The significant MS operating parameters were: ionization voltage, 70 eV; ion source temperature 230^o^C; mass range 50–600 U (Adams, 2001).

GC-MS was performed for the *n*-hexane fraction of the ethanol extract of *C. aciculatus*. The constituents were identified by comparison of their mass spectra with reference spectra in the computer library (NIST 05).

### Statistical Analysis

All the results were expressesd as mean ± SEM. One-way and two-way ANOVA followed by Bonferroni’s test was performed for statistical analysis. The results were considered significant when *p* < 0.05. Graphpad Prism 5.03 software was used for conducting statistical analysis.

### Computational Study

Computational study of the identified compounds was conducted against human COX-2 enzyme, which is the inducible enzyme in human body contributing to inflammatory pain. Human COX-2 or Prostaglandin G/H Synthase 2 (PGH_2_) is a homo-dimer protein comprising 604 amino acids in each monomeric unit (UniProtKB ID: P35354). Homology model of the human COX-2 was built using Easy Modeler 4.0 taking three reported models as templates (PDB ID: 5F1A_A, 5IKQ_A, and 5F19_A) (Kuntal et al., 2010). The initial model was selected based on dope (Discrete Optimized Protein Energy) score and further validated by analyzing Ramachandran plot (Using procheck and rampage) (Laskowski, 2001; Lovell et al., 2003). Knowledge based energy calculation was performed in ProSA (Wiederstein and Sippl, 2007). All the selected ligands were prepared using Autodock tools and the binding site in the enzyme was predicted through ProBiS server (Konc et al., 2015). Initial docking grid (40 × 40 × 40, 0.7 Å) enclosing the active binding site was generated depending on the binding of arachidonic acid. Finally all the prepared ligands were docked into the predicted binding pocket of the validated homology model of COX-2 utilizing Autodock Vina (Trott and Olson, 2010).

## Results

### Results of Identification of Phytochemical Constituents

Preliminary phytochemical investigation of the ethanol extract of *C. aciculatus* indicated the presence of tannins, terpenoids, alkaloids, flavonoids, saponins and glycosides.

### Results of Acute Toxicity Test

In the acute toxicity study, no mortality was observed even at the highest doses of the extract. As per the Globally Harmonized Classification System (GHS) of chemicals, the crude extract of *C. aciculatus* can be categorized as category 5 or unclassified i.e., LD_50_ more than 5,000 mg/kg b.w. Conventionally, experimental doses should be ten times less than that of the toxic dose to avoid any interference of the toxicity in the observed pharmacological activity. Although no toxicity was observed in the acute toxicity study, test doses (500 and 750 mg/kg) in the present experiment were kept around ten times below the highest dose of acute toxicity test.

### Results of Acetic Acid Induced Writhing Test

Ethanol extract of *C. aciculatus* significantly (*p* < 0.05) reduced the number of writings in the test mice at the both doses of 500 and 750 mg/kg tested (**Table 1**). Both the results were comparable with diclofenac sodium (25 mg/kg) treated group, which showed the most potent analgesic activity.

**Table 1:** Effect of *C. aciculatus* extract on acetic acid induced writhing in mice.

Treatment Groups (*n* = 5)	Dose (mg/kg b.w.)	Number of writhing
Control (1% Tween 80 in water)	10 ml/kg	31.2 ± 1.49
Diclofenac sodium	25	8.4 ± 0.51^∗∗^
*C. aciculatus* extract	500	20.2 ± 0.86^∗^
	750	16.8 ± 1.07^∗∗^


*Values are represented as mean ± SEM, ^∗^*p* < 0.05 vs. control, ^∗∗^*p* < 0.005 vs. control.*

### Results of Hot Plate Test

In hot plate test, *C. aciculatus* extract displayed analgesic effect by increasing the response time in test mice at a dose and time dependent manner (**Table 2**). The analgesic effect rose over time to the peak at 90 min and decreased afterwards. All the results were tested significant when compared to the standard morphine.

**Table 2:** Effect of *C. aciculatus* extract in hot plate test in mice.

Treatment Groups (*n* = 5)	Dose (mg/kg)	Reaction time (sec)				
		
		0 min	30 min	60 min	90 min	120 min
Control (1% Tween 80 in water)	10 ml/kg	4.20 ± 0.09	4.02 ± 0.165	4.01 ± 0.205	4.01 ± 0.201	4.02 ± 0.196
Morphine	25	4.24 ± 0.08	7.5 ± 0.15^∗∗^	12.6 ± 0.22^∗∗^	10.62 ± 0.18^∗∗^	8.31 ± 0.27^∗∗^
*C. aciculatus* extract	500	4.12 ± 0.16	5.33 ± 0.28^∗^	6.72 ± 0.19^∗∗^	6.49 ± 0.23^∗∗^	5.23 ± 0.08^∗∗^
	750	4.37 ± 0.06	6.63 ± 0.20^∗∗^	7.72 ± 0.20^∗∗^	6.65 ± 0.29^∗∗^	5.44 ± 0.19^∗∗^


*Values are represented as mean ± SEM, ^∗^*p* < 0.05 vs. control, ^∗∗^*p* < 0.005 vs. control.*

### Results of LC-MS Analysis

The chemical constituents previously reported from *C. aciculatus* were matched with the molecular ion peaks observed in the recorded LC-MS (**Supplementary Figure S1**). According to molecular weights three compounds were suggested as aciculatin, nudaphantin and 5α, 8α-epidioxyergosta-6, 22-diene-3β-ol or ergosterol peroxide having retention time of 34.859, 35.545, and 39.993 min, respectively (**Table 3**). Another eight compounds from different plants belonging to the Poaceae family were also suggested to be present in *C. aciculatus* by analyzing the mass spectra (**Table 3**).

**Table 3:** Compounds identified in the ethanol extract of *C. aciculatus* using LC-MS.

	RT (min)	[M + H]^+^ experimental	Molecular formula	Molecular mass Calculated	Suggested compound	Reference
From previously reported compounds	21.5	415.30	C_22_H_22_O_8_	414.41	Aciculatin	Lai et al., 2012
	21.7	391.28	C_21_H_26_O_7_	390.432	Nudaphantin	Su, 2012
	29.3	429.37	C_28_H_44_O_3_	428.657	5α,8α-epidioxyergosta-6,22-diene-3β-ol/Ergosterol peroxide	Hu and Zheng, 2006
From Poaceae family	1.72	571.26	C_30_H_18_O_12_	570.462	Aurofusarin	Merhej et al., 2012
	1.79	603.34	C_40_H_58_O_4_	602.9	Gamma-oryzanol	Manosroi et al., 2012
	2.17	317.18	C_16_H_12_O_7_	316.265	Isorhamnetin	Mika et al., 2005
	2.25	331.28	C_17_H_14_O_7_	330.29	Tricin	Duarte-Almeida et al., 2007
	31.54	279.15	C_18_H_30_O_2_	278.436	9,12,15-Octadecatrienoic acid	Krishna et al., 2013
	35.81	297.23	C_12_H_12_N_2_O_3_S_2_	296.37	*([(4-Oxo-3,5,6,7-tetrahydro-4*H*-cyclopenta[4,5]thieno[2,3-*d*]pyrimidin-2-yl)methyl]thio)acetic acid*	Krishna et al., 2013
	35.95	473.34	C_31_H_52_O_3_	472.41	Campesterol	Prinsen et al., 2012
	41.14	469.32	C_32_H_52_O_2_	468.766	Lupeol acetate	Varghese et al., 2009


### Results of GC-MS Analysis

Gas chromatography of the *n*-hexane fraction of the test extract led to the elution of three compounds (**Supplementary Figure S2**). Through searching the database for known fragmentation patterns, these three compounds were identified as citronellylisobutyrate (18.58%), 2,4-dihydroxy-7-methoxy-(2H)-1,4-benzoxazin-3(4H)-one (DIMBOA) (4.57%) and nudaphantin (76.85%) (**Table 4**).

**Table 4:** Chemical constituents identified in the *n*-hexane fraction of *C. aciculatus* extract by GC-MS.

Peak no.	RT	%Area	%Height	A/H	Compound analyzed	Probable molecular weight	Probable molecular formula
**1.**	36.89	18.58	11.81	8.54	Citronellyl Isobutyrate	226	C_14_H_26_O_2_
**2.**	38.21	4.57	4.47	5.53	2,4-dihydroxy-7-methoxy-(2H)-1,4-benzoxazin-3(4H)-one (DIMBOA)	211.17	C_9_H_9_NO_5_
**3.**	53.94	76.85	83.72	4.98	Nudaphantin	390.42	C_21_H_26_O_7_


## Results of Computational Study

**FIGURE 1:**
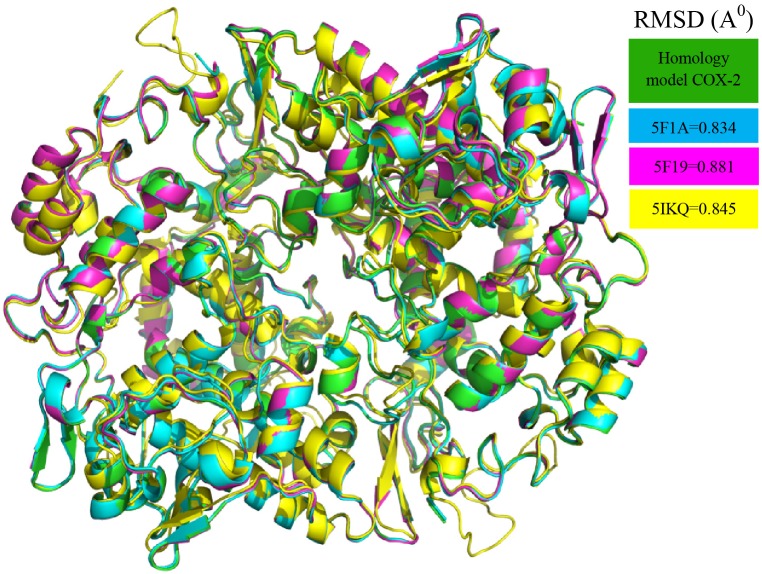
Homology model of Human COX-2 enzyme.

The initial homology model of human COX-2 enzyme was selected based on the lowest dope profile and used for the docking (**Figure 1**). The sequence alignment is given in the **Supplementary Materials** (**Supplementary Figure S3**). The Ramachandran plot analysis showed that the number of residues in the favored, allowed and outlier regions are 97.8, 2.2, and 0.0%, respectively. In addition to the Z-score of -7.48 in knowledge base energy calculation, the low RMSD (root-mean-square deviation) of 0.8 to 0.9 Å between the overlaid model and template structure implied that the homology model was a theoretically reliable for molecular modeling study (**Supplementary Figure S4**). The homo-dimer model of COX-2 was generated from its monomeric units using GalaxyGemini following energy minimization step. The arachidonic acid binding pocket was identified with the following amino acid residues: Ala513, Val335, Gly512, Ser516, Tyr341, Phe343, Val102, Met99, Leu103, Val509, Leu338, Tyr371, and Ser339 (**Supplementary Figure S5**). Fourteen ligands obtained from the LC-MS and GC-MS analysis were docked into the active site of the human COX-2 homology model and nine different poses of the ligands in the binding site were analyzed (**Supplementary Figures S6**–**S18**) and the results are presented in **Table 5**. Six ligands, namely citronellyl isobutyrate, octadeca 9,12,15-trienoic acid, ([(4-oxo-3,5,6,7-tetrahydro-4Hcyclopenta[4,5] thieno [2,3-d]pyrimidin-2-yl)methyl] thio) acetic acid, campesterol, gamma oryzanol and tricin were found to interact with some amino acid residues at the catalytic site with hydrophobic interactions. Eleven out of thirteen compounds showed higher affinity for COX-2 than that of arachidonic acid (-6.4 kcal/mol) with the highest affinity observed for aciculatin and aurofusarin (-9.2 and -11.7 kcal/mol).

**Table 5:** Results of docking studies of the compounds identified in *C. aciculatus* extract on COX-2 enzyme.

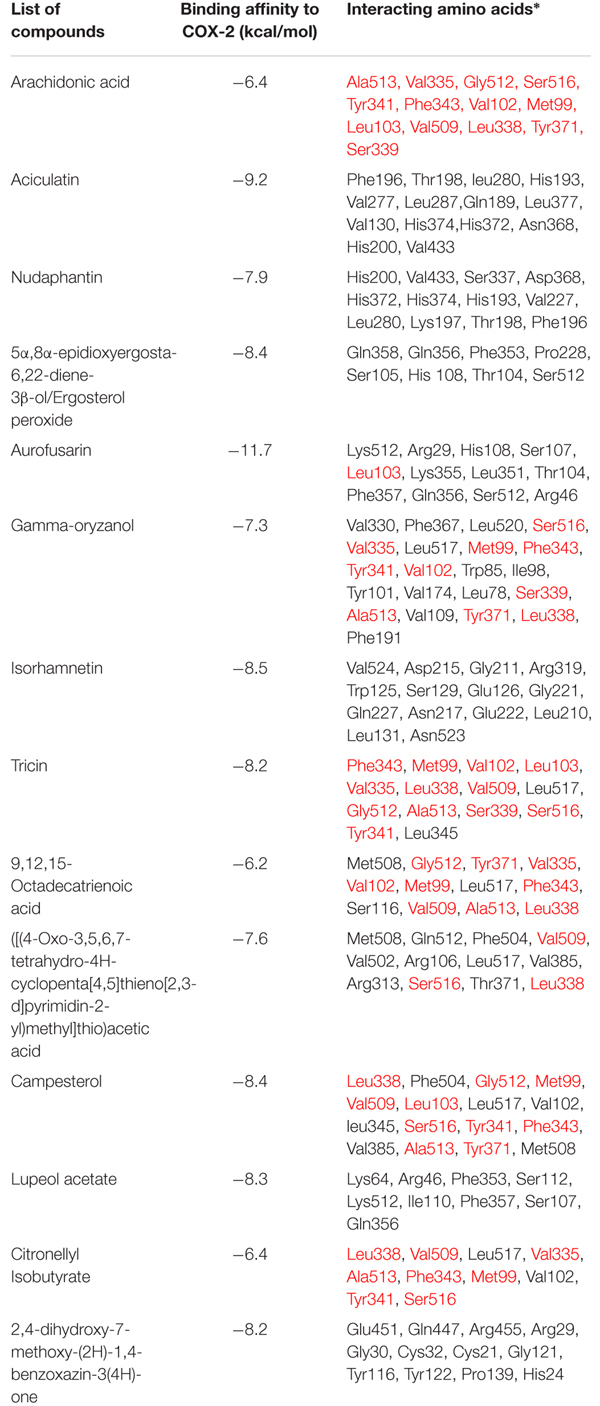

*^∗^Red colored texts indicate amino acid residues involved in the binding of arachidonic acid.*

## Discussion

Acetic acid induced writhing reflex model in mice is a widely accepted and effective pain model for evaluating peripherally acting analgesics (Zulfiker et al., 2010). In this method, acetic acid induces pain sensation via localized inflammatory response resulting from local release of arachidonic acid and subsequent prostaglandins through cyclooxygenase pathway of the arachidonate cascade (Duarte et al., 1988). In other words, peritoneal administration of acetic acid raises the level of PGE_2_ and PGF_2α_ in the peritoneal fluid and causes inflammatory pain (Zulfiker et al., 2010). In our study, ethanol extract of *C. aciculatus* significantly reduced the number of writhing in mice model and this decrease suggests that the test extract showed its analgesic activity through peripheral mechanism, which is the inhibition of prostaglandin biosynthesis by acting on visceral receptors sensitive to acetic acid (Sheikh et al., 2016). It is in agreement with the study reporting the presence of anti-inflammatory principle in this plant (Hsieh et al., 2011).

The hot plate method is a suitable assay for neurologic pain in evaluating centrally acting analgesics. This method measures a complex response to an acute and non-inflammatory nociception. Centrally acting analgesics like opioids usually act via supra spinal and spinal receptors; and therefore can increase the reaction time in hot plate test (Sabina et al., 2009; Mondal et al., 2014). Morphine, used as the standard drug in this test, is a renowned central analgesic that exerts its effect through binding with the opioid receptors (μ, κ, and δ) located in the post and pre synaptic membrane (Mondal et al., 2014). It was demonstrated that oral administration of *C. aciculatus* extract prolonged the response latency period to heat stimulus in test mice that increased at higher dose. The effect was observable from 30 min of treatment and declined at the end of the observation period (**Table 2**). As compared to the standard drug morphine, it can be suggested that the test extract has a mild central analgesic activity.

Results suggested that *C. aciculatus* has potential analgesic activity which is comparable to the standards, *i.e.*, diclofenac sodium and morphine, and showed significant difference from the control. In both bioassays the extract showed positive dose dependent effect confirming that this plant has both peripheral and central analgesic activity. As per the results obtained in preliminary phytochemical screening, the ethanolic extract of *C. aciculatus* contains several important phytoconstituent classes including flavonoids, tannins, and alkaloids. There are several reports available that demonstrate analgesic and anti-inflammatory activity of different compounds from the stated classes (Pathak et al., 1991; Duke, 1992; Bittar et al., 2000; Bello-Ramírez et al., 2003; Wang et al., 2009; Chen et al., 2012). We also identified a number of compounds in the test extract through LC-MS and GC-MS that were previously reported from this plant and the Poaceae famly. Among these compounds, the anti-inflammatory activity of aciculatin is well established. Aciculatin inhibits the expression of cyclooxygenase-2 (COX-2) by suppressing NF-κB and JNK/p38 MAPK activation pathways (Hsieh et al., 2011). COX-2 is the inducible isoenzyme in the arachidonate cascade that is responsible for inflammatory pain (Sinatra, 2002). Thus inhibition of its expression will lead to pain inhibition. Moreover, this plant contains quercetin and daucosterol (Hu and Zheng, 2006). Quercetin acts as an analgesic both in neurogenic and inflammatory phases (Filho et al., 2008), whereas daucosterol reduces local inflammation (Mavar-Manga et al., 2008). So, these constituents along with aciculatin can be held responsible for the observed antinociceptive activity of *C. aciculatus*.

Molecular docking studies with the identified ligands against human COX-2 enzyme revealed that, among the thirteen phytoconstituents, six compounds interacted with a number of amino acid residues (Ala513, Val335, Gly512, Ser516, Tyr341, Phe343, Val102, Met99, Leu103, Val509, Leu338, Tyr371, Ser339) associated with the binding of arachidonic acid at the catalytic site, with binding affinity ranging between -6.2 and -8.4 kcal/mol. Among these six compounds, tricin, an *O*-methylated flavone, showed lower binding affinity (-8.2 kcal/mol) than that of aciculatin and aurofusarin, but with greater number (12) of interaction with the amino acid residues involved in the binding of arachidonic acid through hydrophobic interactions. Campesterol interacted with Leu338, Gly512, Met99, Val509, Leu103, Ser516, Tyr341, Phe343, Ala513 and Tyr371 with an affinity of -8.4 kcal/mol. Gamma oryzanol also interacted with 10 amino acid residues with an affinity of -7.3 kcal/mol. 9, 12, 15-octadecatrienoic acid (Binding affinity: -6.2 kcal/mol) and citronellyl isobutyrate (Binding affinity: -6.4 kcal/mol) showed interaction with nine and eight amino acid residues, respectively. The other seven compounds also showed some affinity for COX-2 enzyme but the amino acid residues were not involved in the binding of arachidonic acid. So the conclusion can be drawn from these results that, these identified secondary metabolites may have some part in the observed analgesic activity of this plant extract.

## Conclusion

Results from the present study confirmed that *C. aciculatus* possesses significant and dose dependent analgesic activity, which supports the uses of the plant in folk medicine. This activity may be attributed to aciculatin, an anti-inflammatory glycosylflavone, identified in the plant and the identified phytoconstituents showing promising affinity toward human COX-2 enzyme in computational study.

## Ethics Statement

This study was carried out in accordance with the internationally accepted principle for laboratory use and care (National Research Council of USA, 1996). The protocol was approved by a Animal Ethics Committee, Pharmacy Discipline, Life Science School, Khulna University, Khulna, Bangladesh (Protocol Number: KU/PHARM/AEC/15/006/025). Three members ethics committee consists of Dr. Ashish Kumar Das, Professor and chairman of the committee (dasasish03@yahoo.com), Dr. Jamil A Shilpi (jamilshilpi@yahoo.com), Professor and Dr. Shaikh Jamal Uddin, Associate Professor (uddinsj@yahoo.com); Pharmacy Discipline, Life Science School, Khulna University, Khulna, Bangladesh.

## Author Contributions

SZ, NB, and NS carried out the extraction and *in-vivo* studies under the supervision of SU, JS, AD, and SS. JS, HH, MI, and RR conducted the phytochemical profiling through LC-MS and GC-MS. SZ, SD, and MR conducted the computational study. SA carried out the statistical analysis. LN, SS, and SU designed the work. SZ and NS drafted the manuscript. EA, LN, and RR revised the manuscript. All authors have approved the manuscript for submission.

## Conflict of Interest Statement

The authors declare that the research was conducted in the absence of any commercial or financial relationships that could be construed as a potential conflict of interest.
