# Supplementary material for: Analgesic Activity, Chemical Profiling and Computational Study on Chrysopogon aciculatus
Source: Front Pharmacol. 2018 Oct 15;9:1164. doi: 10.3389/fphar.2018.01164 (PMC6196237; doi:10.3389/fphar.2018.01164)
Supplement: Supplementary file 1 [file Data_Sheet_1.docx]

**Supplementary Material**

**Analgesic Activity, Chemical Profiling and Computational Study on *Chrysopogon aciculatus***

**S. M. Neamul Kabir Zihad^1γ^, Niloy Bhowmick^1γ^, Shaikh Jamal Uddin^1γ*^, Nazifa Sifat^1^, Md. Shamim Rahman^2^, Razina Rouf^3^, Muhammad Torequl Islam ^4, 5^, Shrabanti Dev^1^, Hazrina Hazni^6^, Shahin Aziz^7^, Eunüs S. Ali^8^,** **Asish K. Das^1^, Jamil A Shilpi^1^, Lutfun Nahar^9^, Satyajit D. Sarker^9*^**

*^1^Pharmacy Discipline, Life Science School, Khulna University, Khulna-9208, Bangladesh*

*^2^Biotechnology and Genetic Engineering Discipline, Life Science School, Khulna University, Khulna-9208, Bangladesh*

*^3^Department of Pharmacy, Faculty of Life Science, Bangabandhu Sheikh Mujibur Rahman Science & Technology University, Gopalganj, Bangladesh*

*^4^Department for Management of Science and Technology Development, Ton Duc Thang University, Ho Chi Minh City-700000, Vietnam*

*^5^Faculty of Pharmacy, Ton Duc Thang University, Ho Chi Minh City-700000, Vietnam*

*^6^Centre for Natural Products and Drug Discovery, University of Malaya, Kuala Lumpur, Malaysia*

*^7^Chemical Research Division, Bangladesh Council of Scientific and Industrial Research, Dhaka, Bangladesh*

*^8^Department of Biochemistry and Molecular Genetics, Northwestern University Feinberg School of Medicine, 320 E Superior St, Chicago, IL 60611, USA.*

*^9^Medicinal Chemistry and Natural Products Research Group, School of Pharmacy and Biomolecular Sciences, Liverpool John Moores University, James Parsons Building, Byrom Street, Liverpool L3 3AF, UK.*

**^γ^**Authors contributed equally

***Correspondance:**

Dr. Shaikh Jamal Uddin

uddinsj@yahoo.com

Dr. Satyajit D. Sarker

S.Sarker@ljmu.ac.uk

**Table S1:** Reported traditional uses, isolated compounds and pharmacological properties of *C. aciculatus*.

| **Scientific name** | **Family** | **Traditional uses** | **Isolated compounds** | **Reported pharmacological activities** | **References** |
| --- | --- | --- | --- | --- | --- |
| *Chrysopogon aciculatus* (Retz.) Trin. | Poaceae | - Fever - Common cold - Swelling - Diarrhea - Poison antidote - Dysuria - Kidney stone - Tonsillitis - Stomach ache - Gastric disorders - Liver pain - Cattle leg swelling - Helminthiasis - Ameliorate pain and paralysis | - **Glycosylflavone**   Aciculatin   - **Sterol**   Daucosterol   - **Flavonol**   Quercetin   - **Germacranolide**   Nudaphantin   - **Flavone**   7-de-O-methylaciculatin, Aciculatinone,  4′-O-glucosyl aciculatin,  8-C-β-D-boivinopyranosyl apigenin | - Anthelmintic - Antibacterial - Anticancer - Anti-arthritic - Anti-inflammatory - Cytotoxic | (Hu and Zheng, 2006; Anisuzzaman et al., 2007; Hsieh et al., 2011; Tangjang et al., 2011; Lai et al., 2012; Neamsuvan et al., 2012; Shen et al., 2012; Shih et al., 2012; Su, 2012; Ruizo, 2013; Peter and Thomas, 2015; Shahnaj et al., 2015; Sohel et al., 2016; Stuart, 2016; Pueblos et al., 2017) |


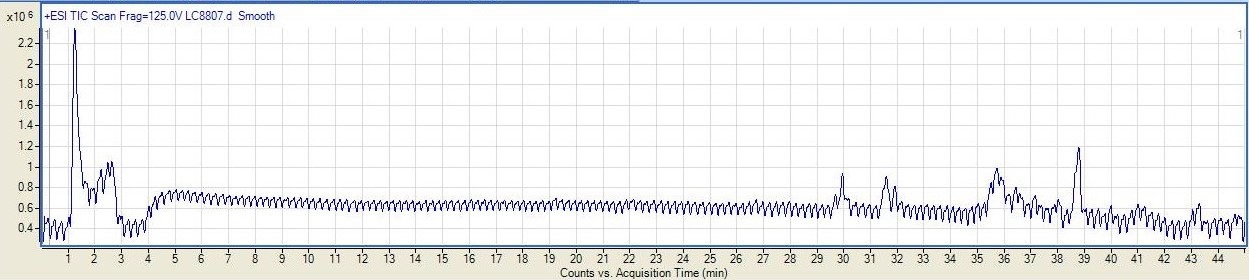


**Figure S1:** LC-MS chromatogram of ethanolic extract of *Chrysopogon aciculatus*.


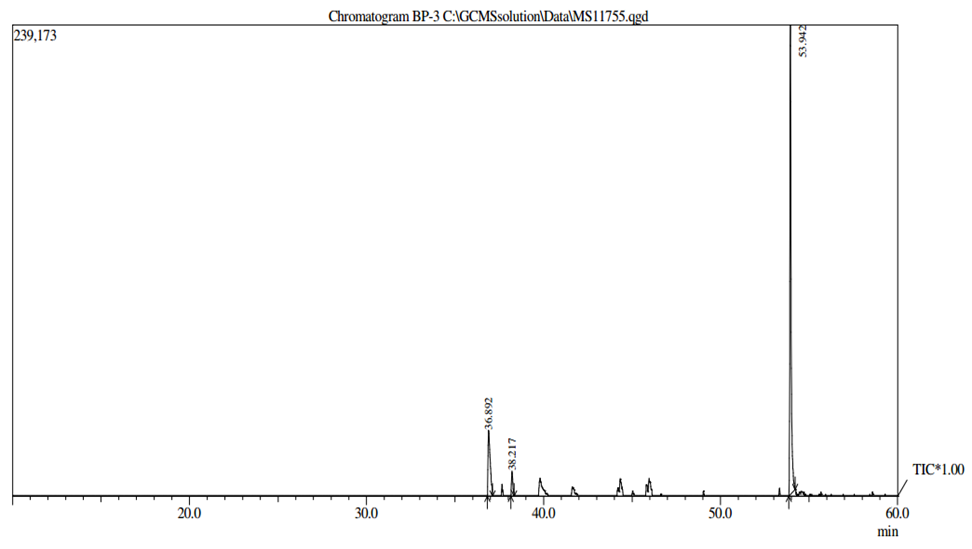
**Figure S2:** GC-MS chromatogram of ethanolic extract of *Chrysopogon aciculatus*.


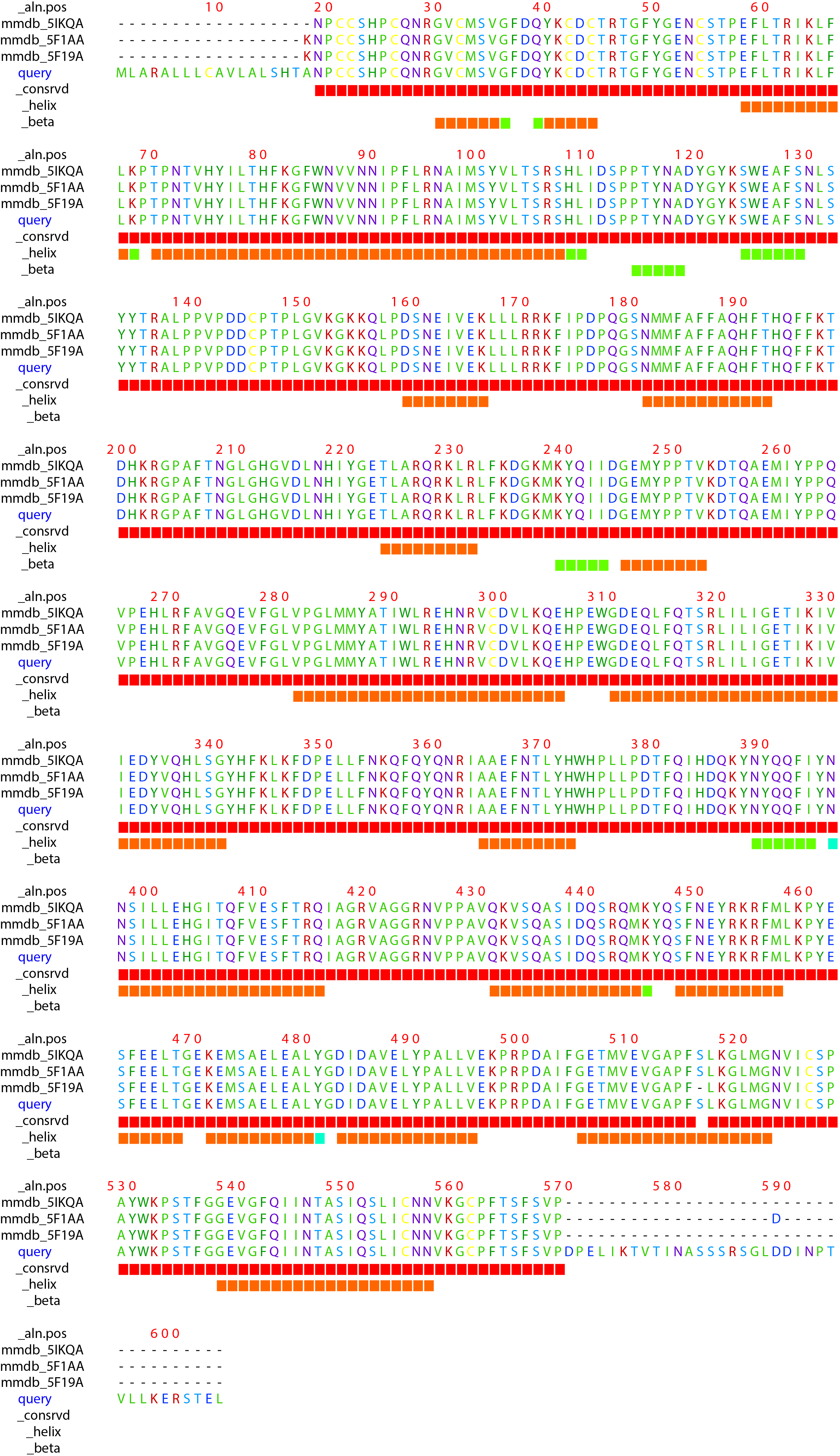


**Figure S3:** Sequence alignment of the constructed homology model with the used template.


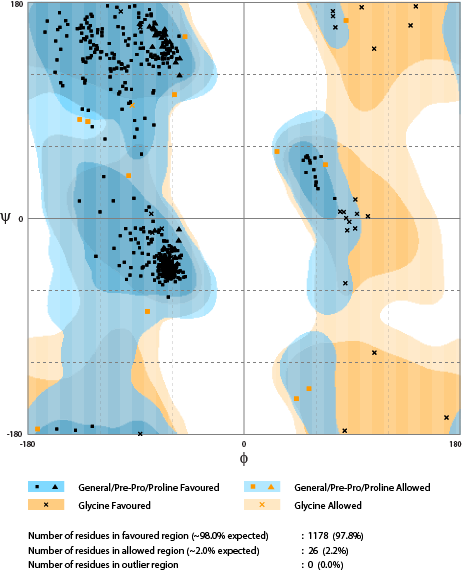

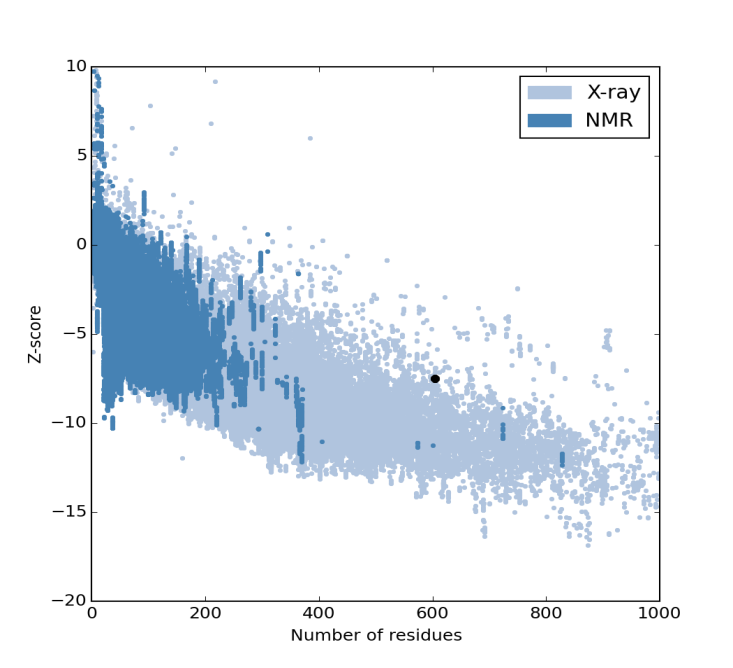

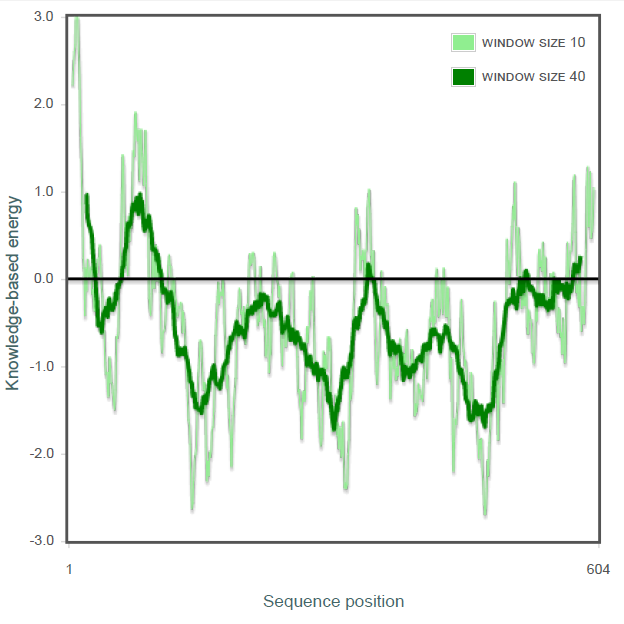


**(A)**

**Z-Score: -7.48**

**(B)**

**(C)**

**Figure S4:** Overall model quality. (A) Ramachandran plot, (B) Z-score, (C) Knowledge based energy.


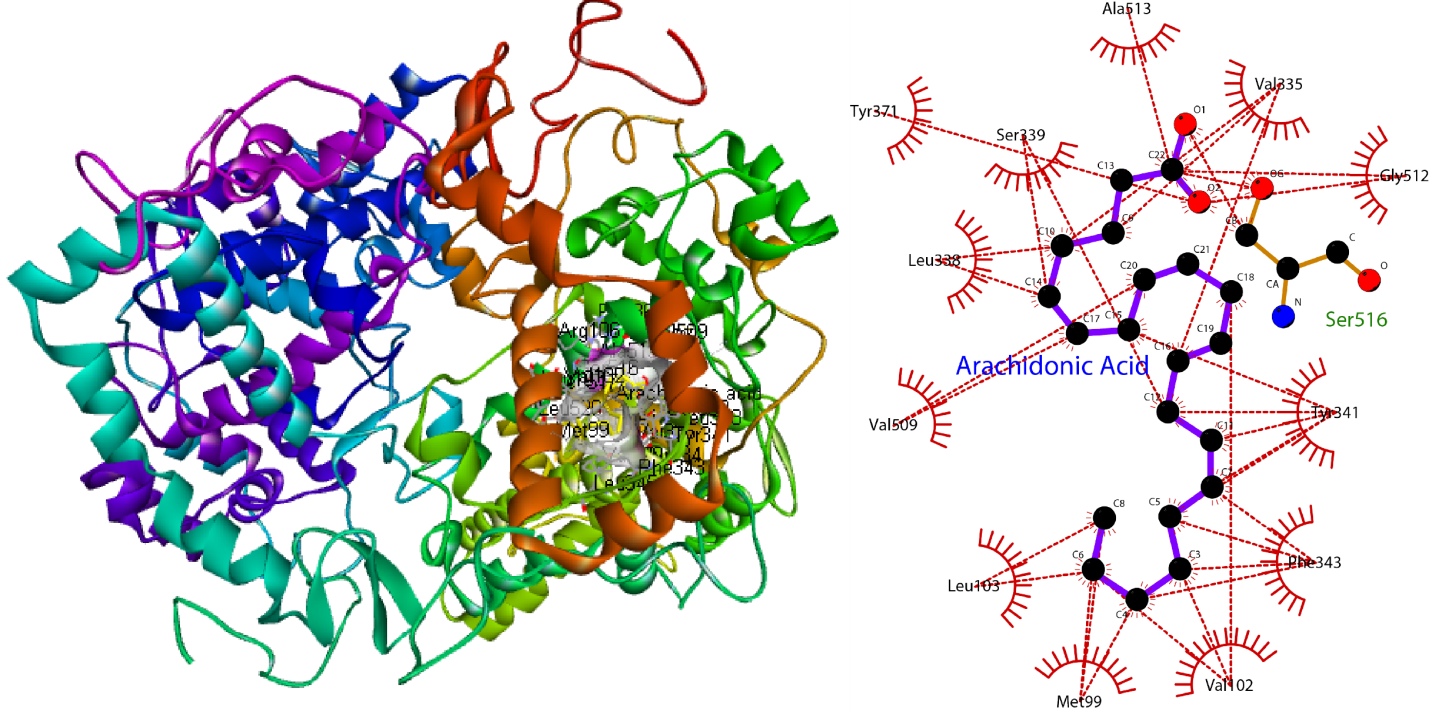


**Figure S5:** Docking of arachidonic acid with human COX-2 enzyme.


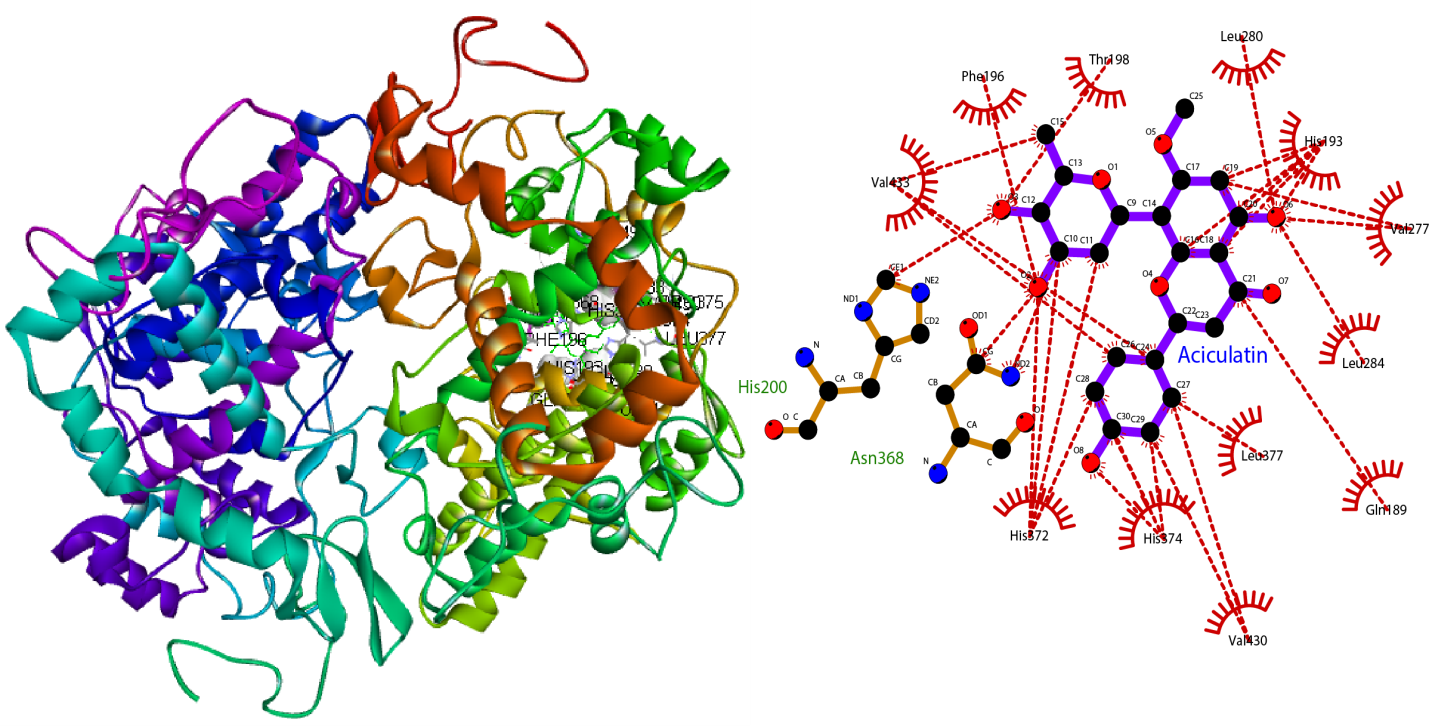


**Figure S6:** Docking of aciculatin with human COX-2 enzyme.


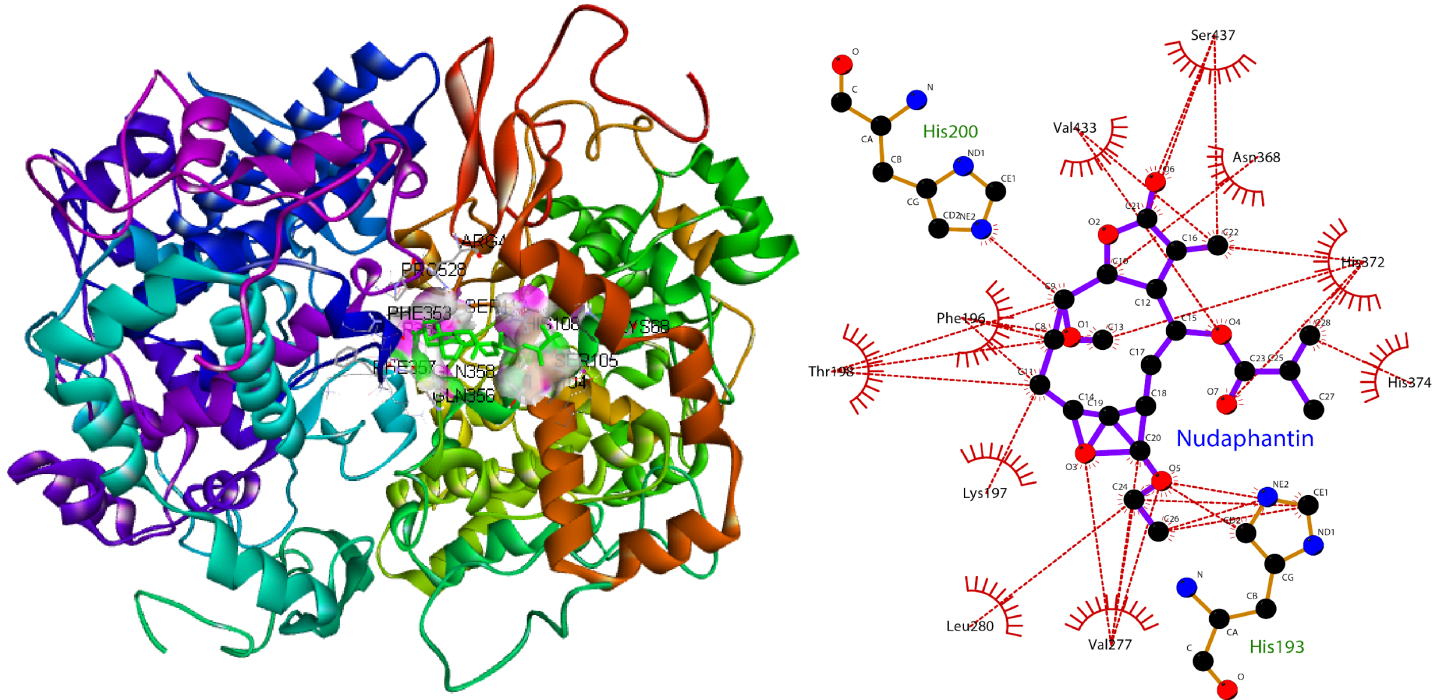


**Figure S7:** Docking of nudaphantin with human COX-2 enzyme.


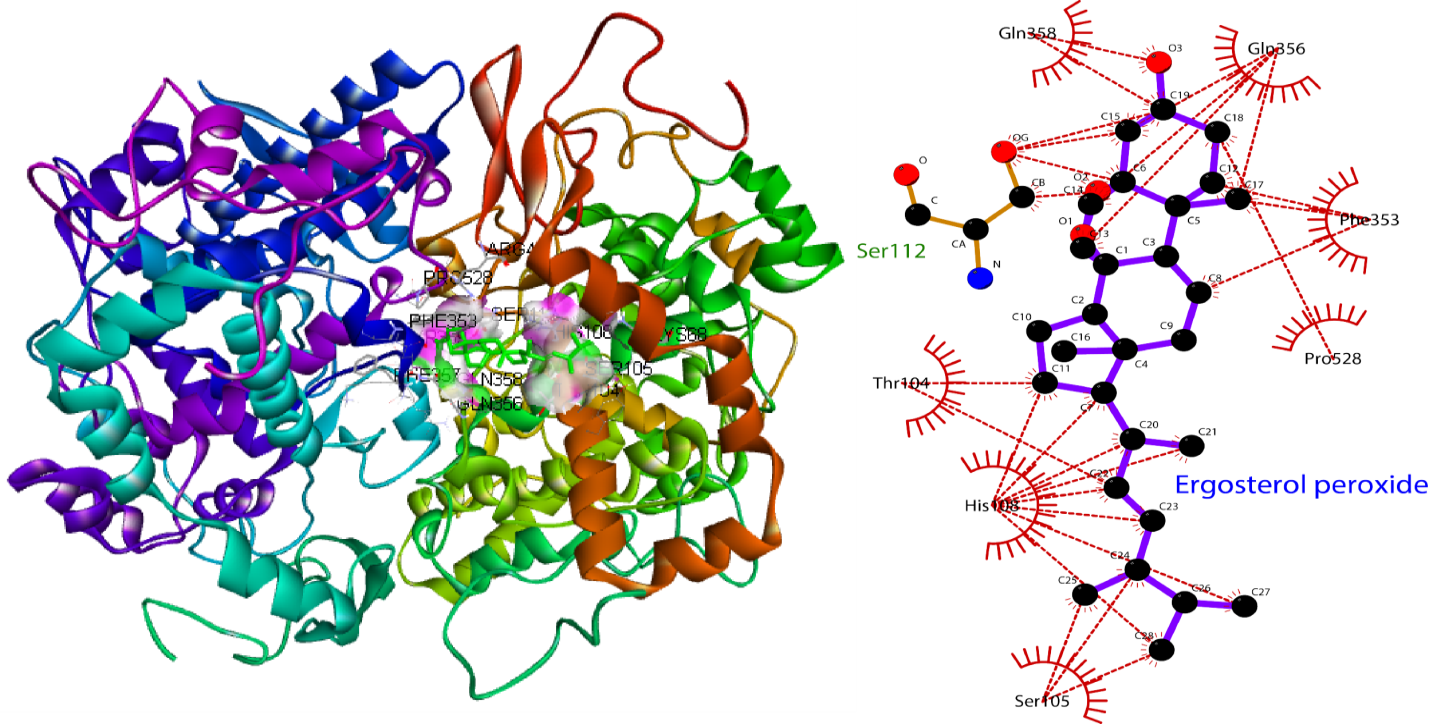


**Figure S8**: Docking of 5α,8α-epidioxyergosta-6,22-diene-3β-ol / ergosterol peroxide with human COX-2 enzyme.


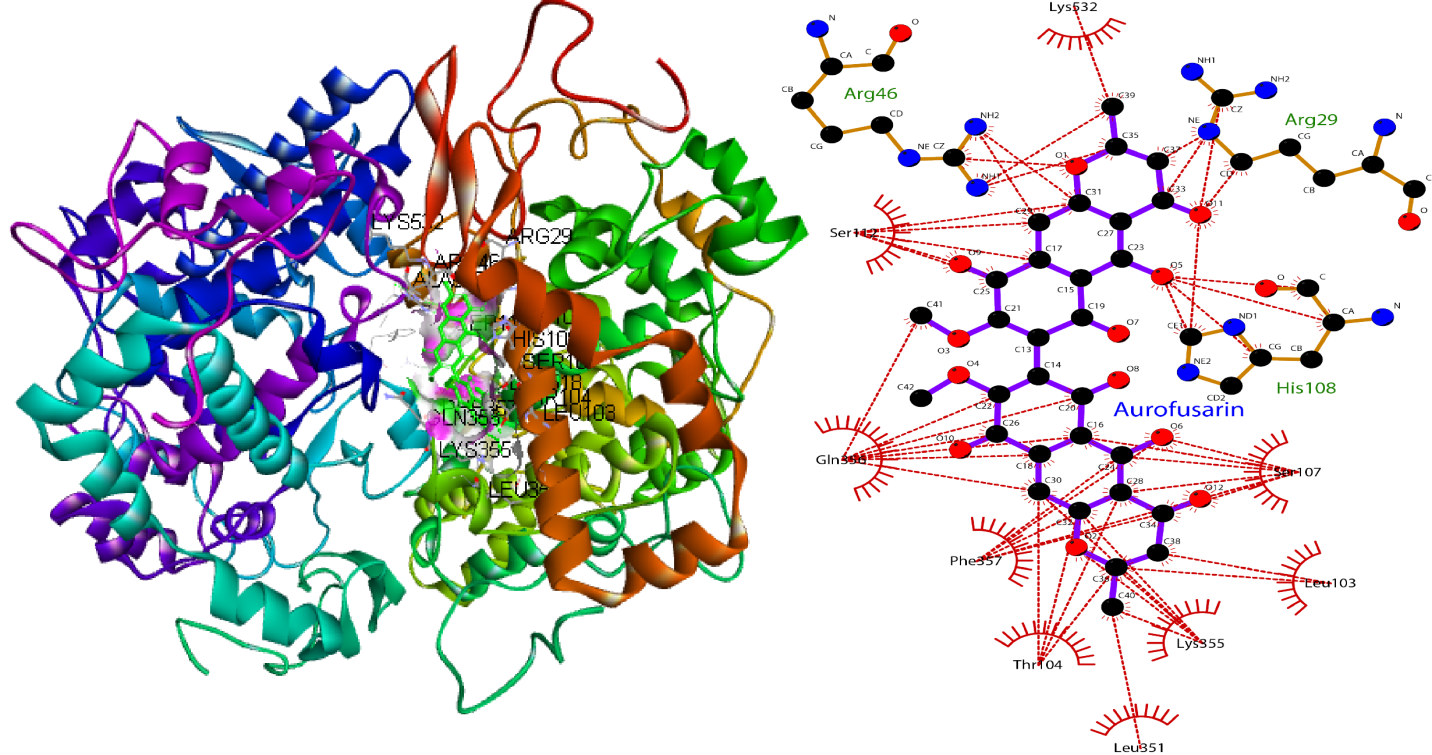


**Figure S9**: Docking of aurofusarin with human COX-2 enzyme.


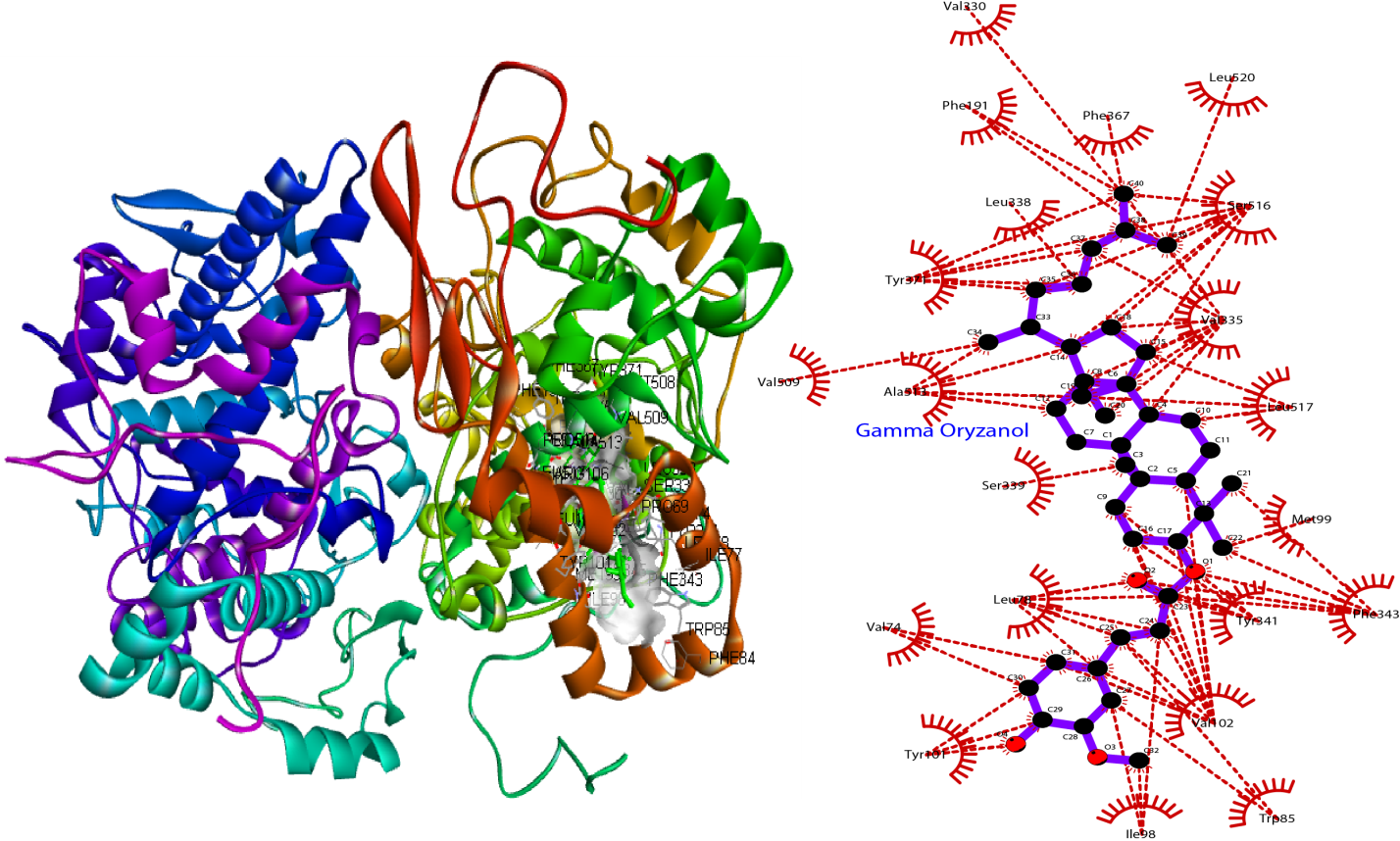


**Figure S10**: Docking of gamma oryzanol with human COX-2 enzyme.


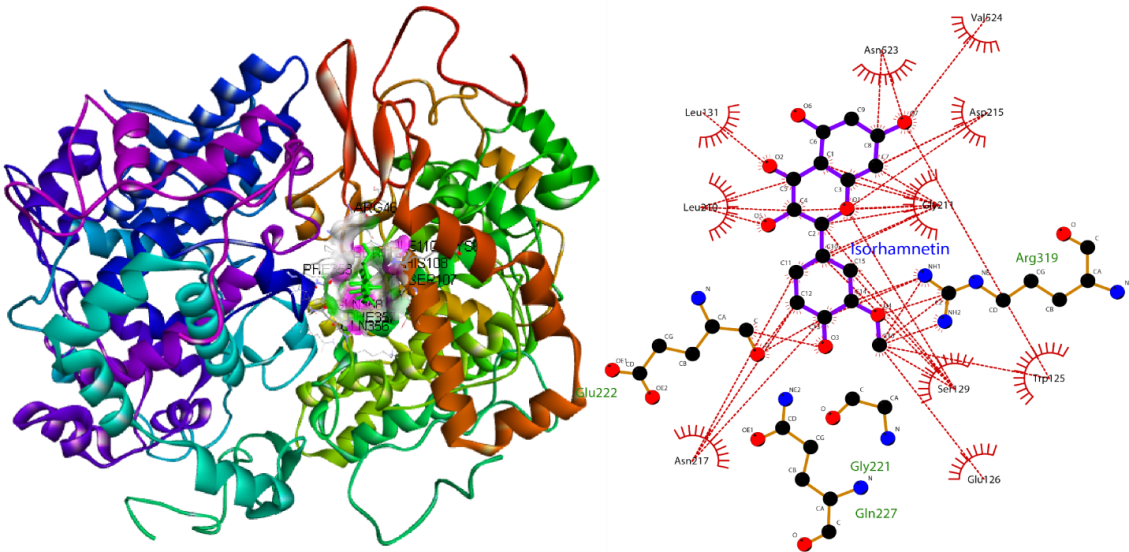


**Figure S11**: Docking of isorhamnetin with human COX-2 enzyme.


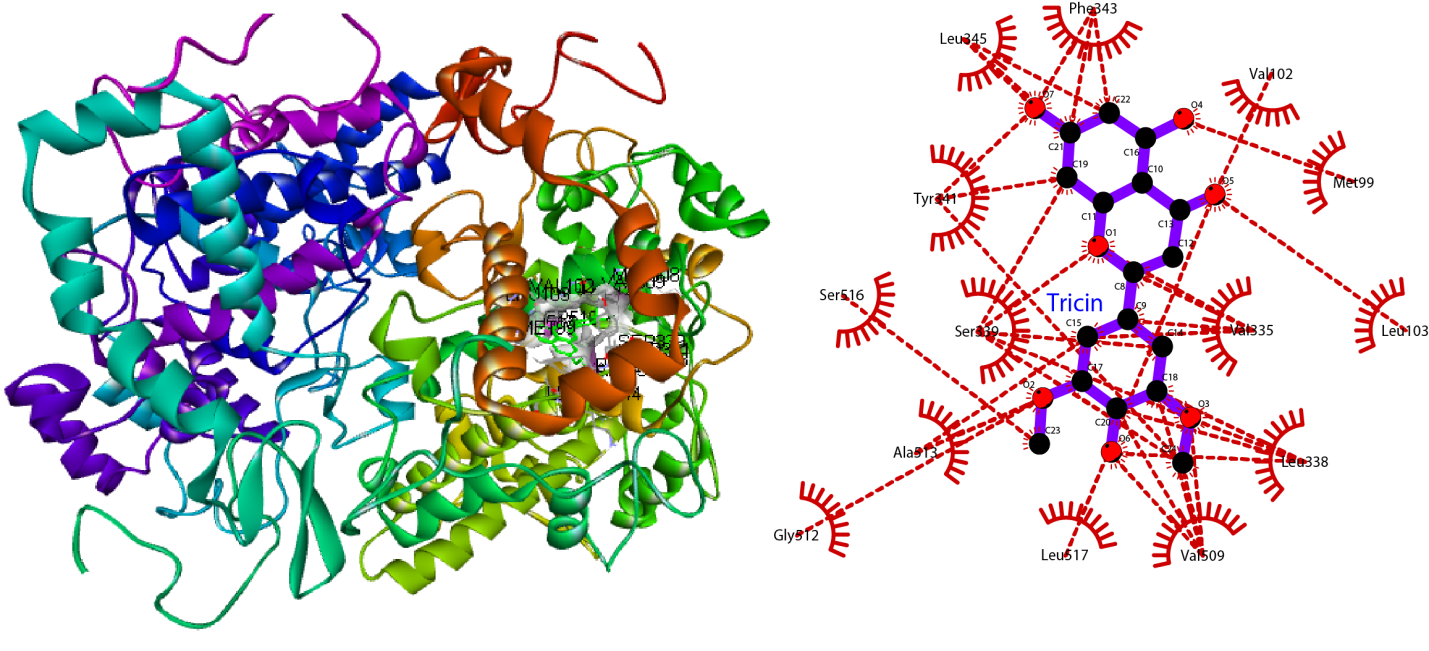


**Figure S12**: Docking of tricin with human COX-2 enzyme.


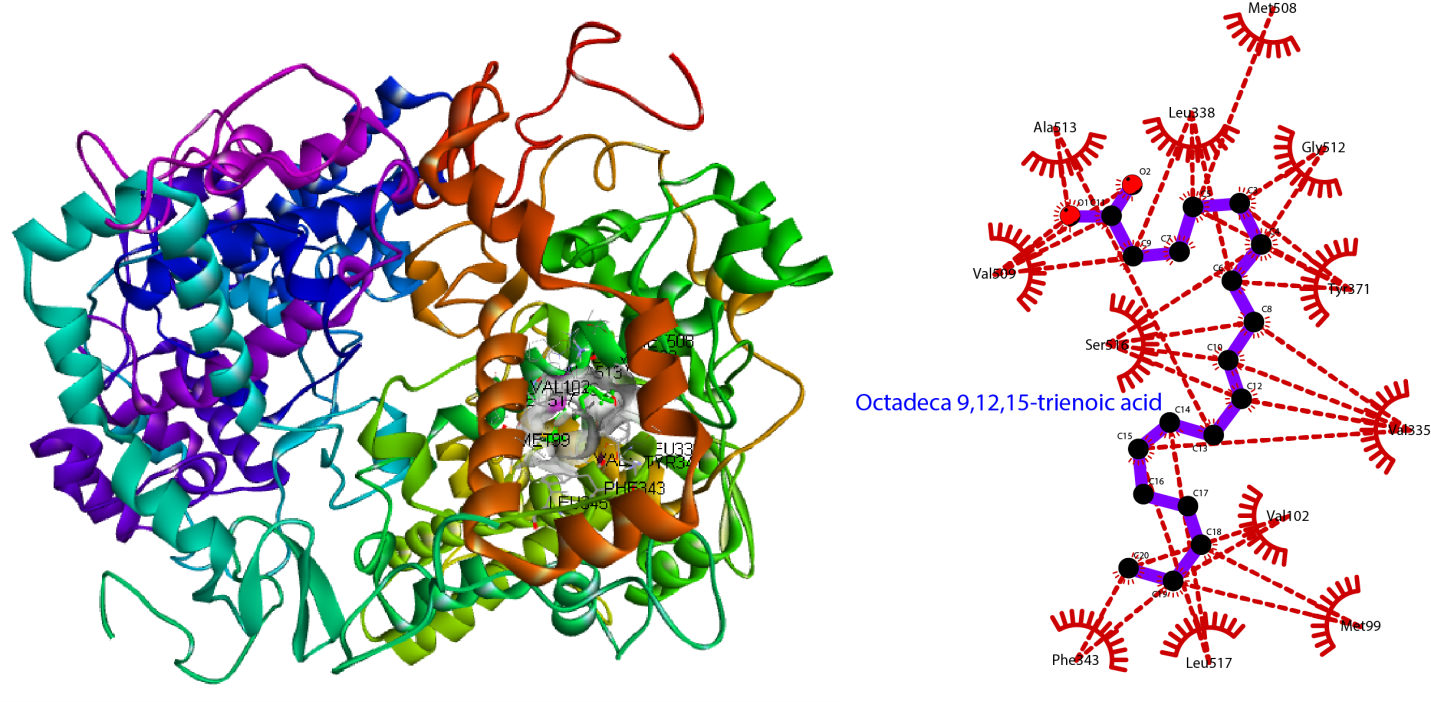


**Figure S13**: Docking of 9,12,15-octadecatrienoic acid with human COX-2 enzyme.


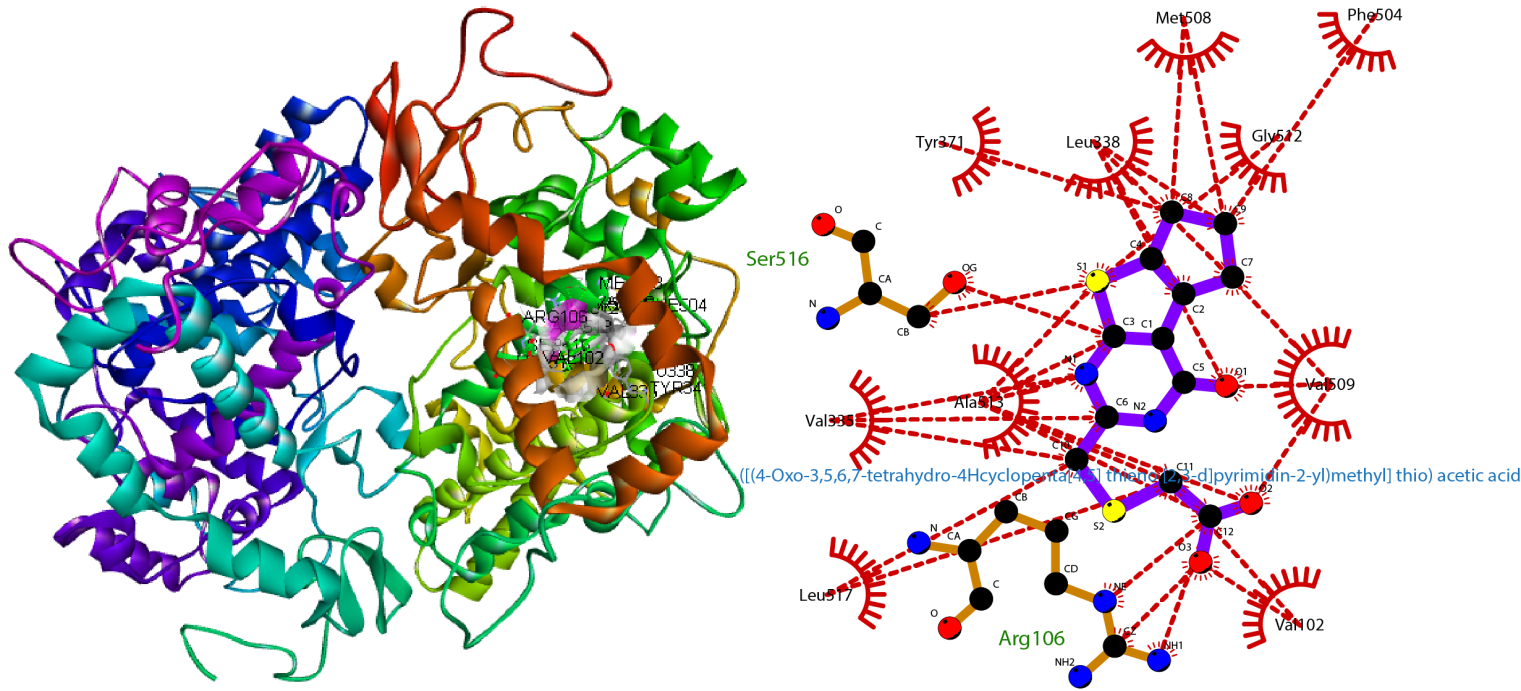


**Figure S14**: Docking of ([(4-Oxo-3,5,6,7-tetrahydro-4*H*-cyclopenta[4,5]thieno[2,3-*d*]pyrimidin-2-yl)methyl]thio)acetic acid with human COX-2 enzyme.


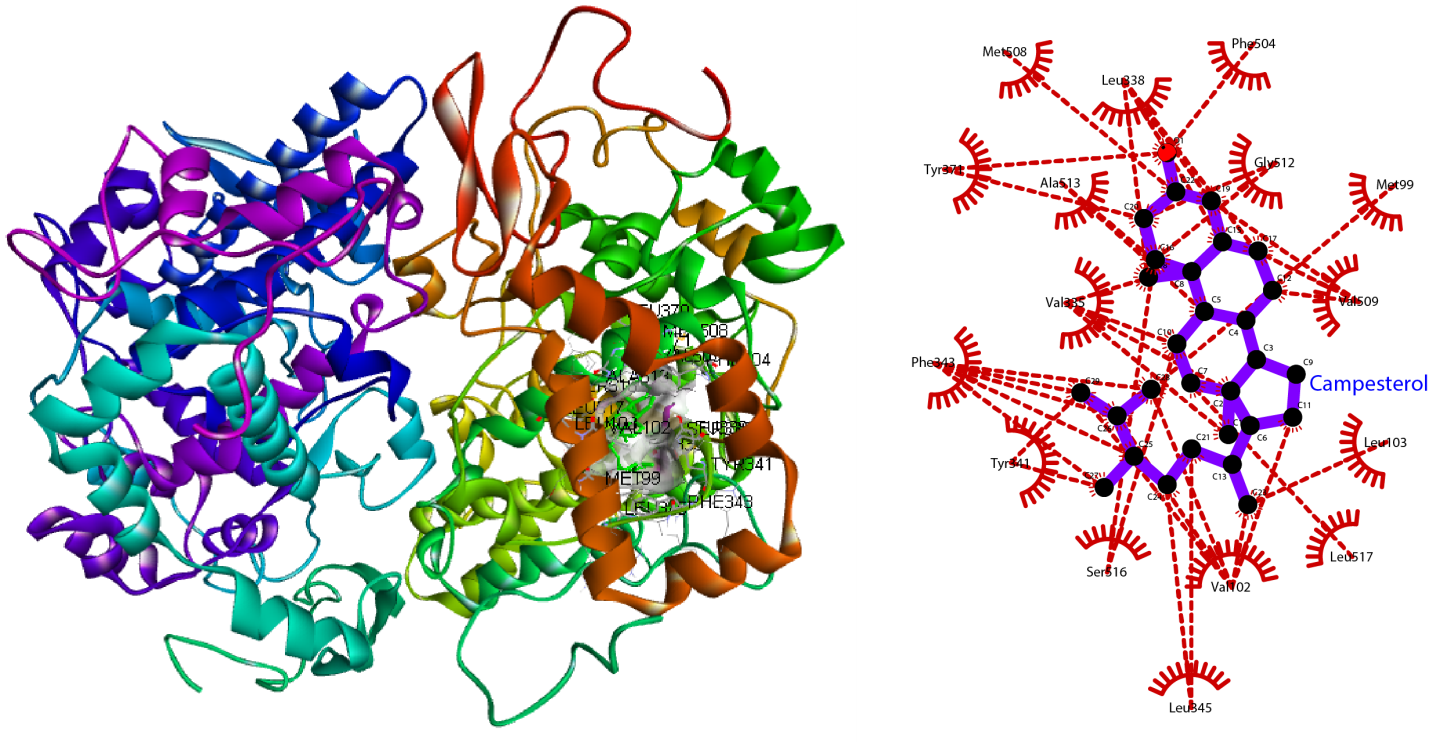


**Figure S15**: Docking of campesterol with human COX-2 enzyme.


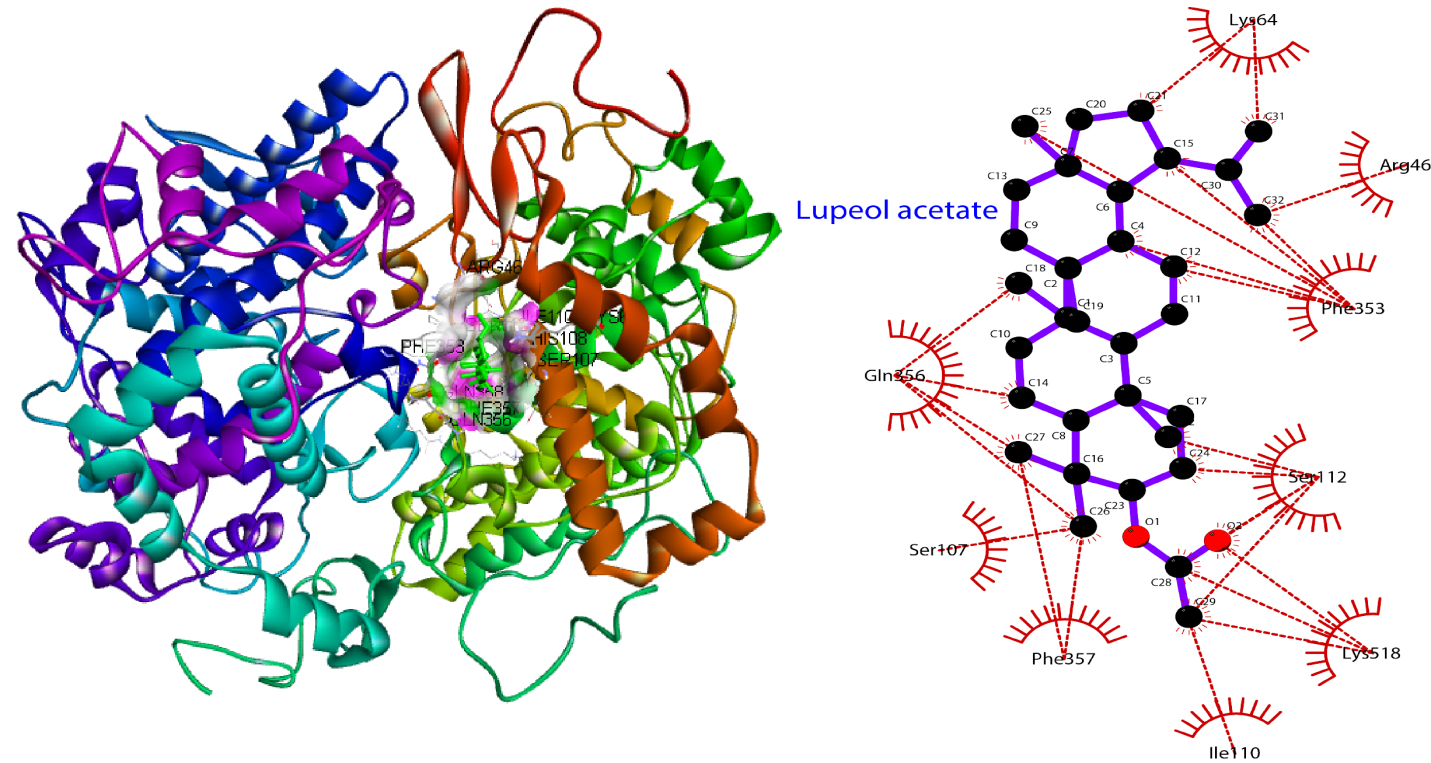


**Figure S16**: Docking of lupeol acetate with human COX-2 enzyme.


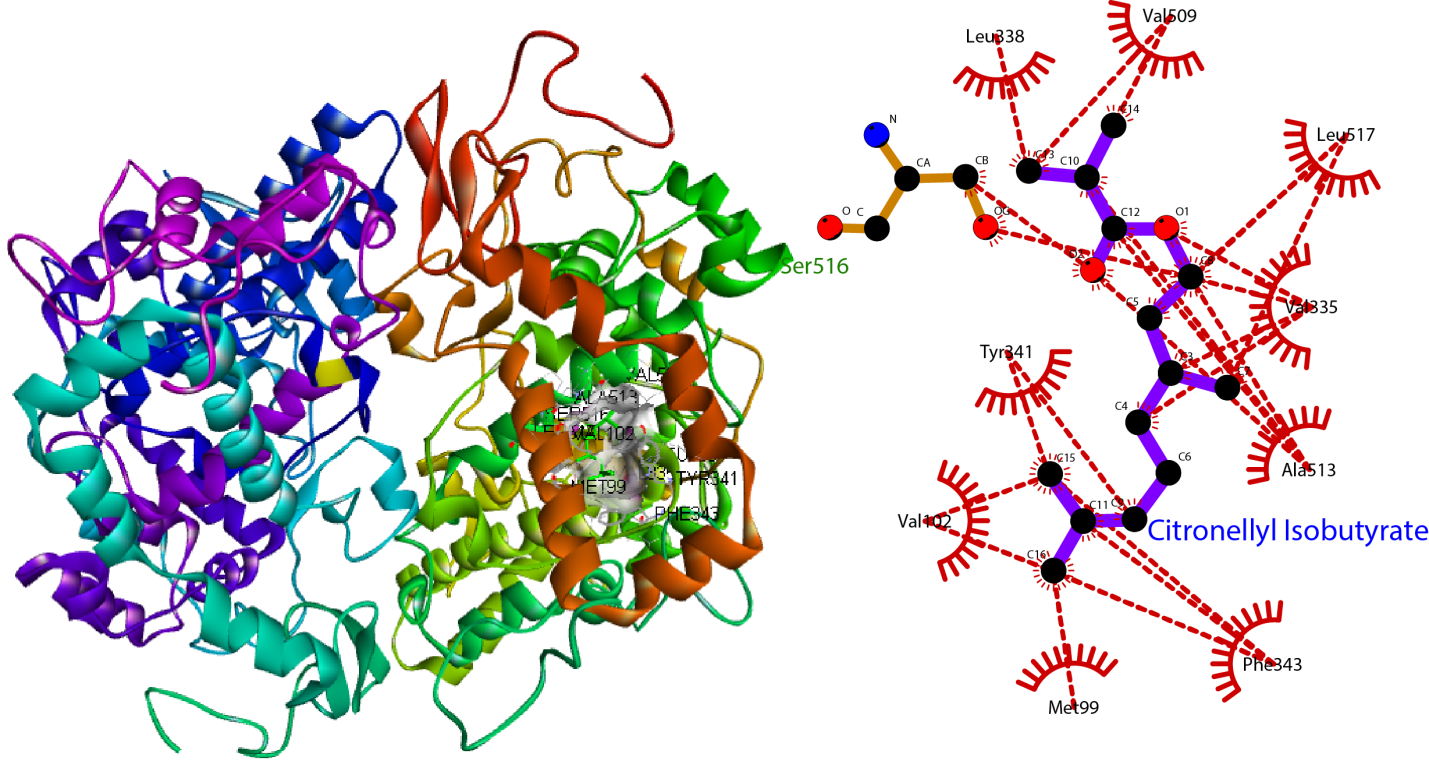


**Figure S17**: Docking of citronellyl isobutyrate with human COX-2 enzyme.


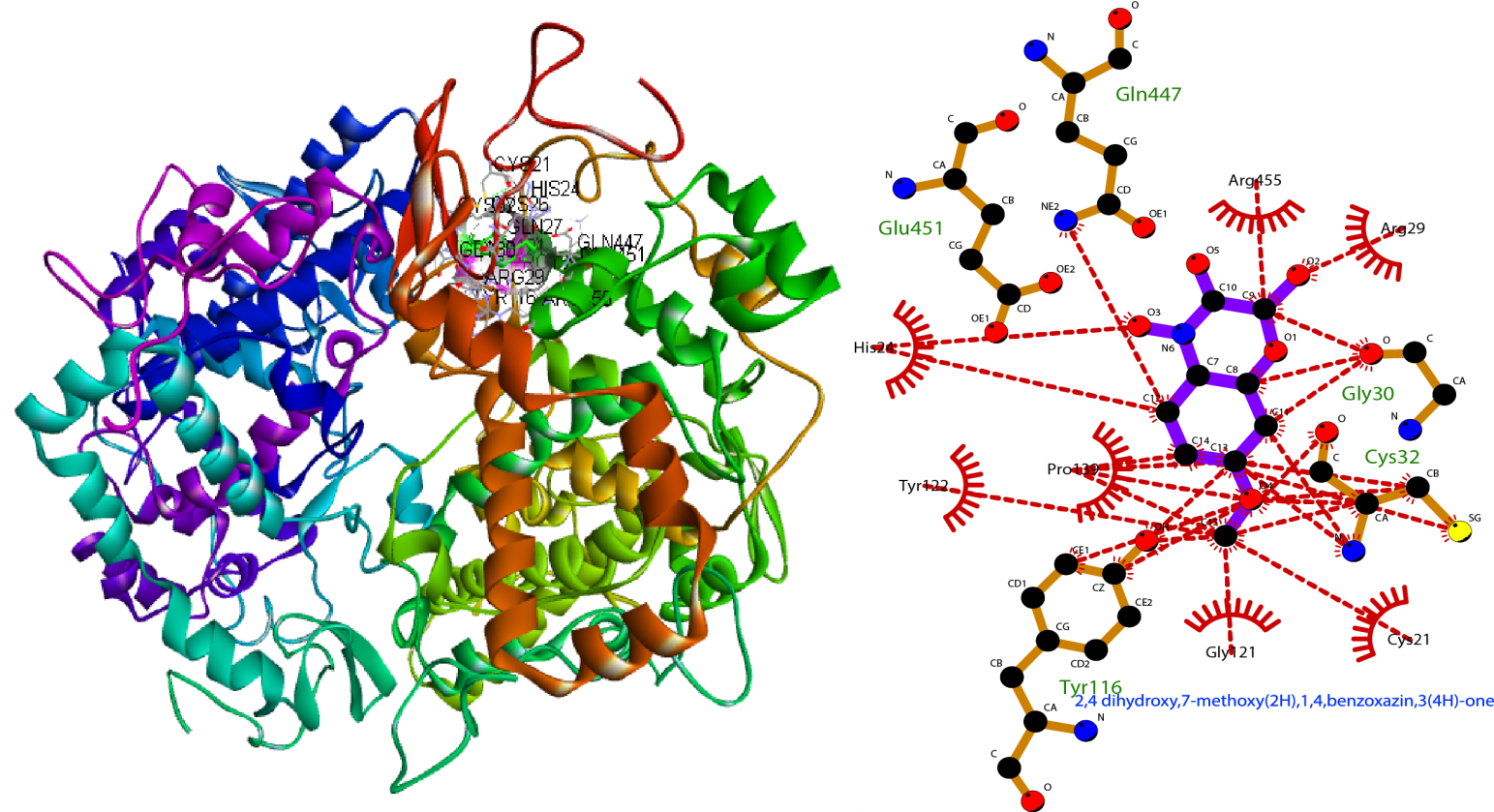


**Figure S18**: Docking of 2,4-dihydroxy-7-methoxy-(2H)-1,4-benzoxazin-3(4H)-one with human COX-2 enzyme.

**References**

Anisuzzaman, M., Rahman, A., Harun-Or-Rashid, M., Naderuzzaman, A., and Islam, A. (2007). An ethnobotanical study of Madhupur, Tangail. *J. Appl. Sci. Res.* 3(7)**,** 519-530.

Hsieh, I.-N., Chang, A.S.-Y., Teng, C.-M., Chen, C.-C., and Yang, C.-R. (2011). Aciculatin inhibits lipopolysaccharide-mediated inducible nitric oxide synthase and cyclooxygenase-2 expression via suppressing NF-κB and JNK/p38 MAPK activation pathways. *J. Biomed. Sci.* 18(1)**,** 28. doi: 10.1186/1423-0127-18-28.

Hu, H.-b., and Zheng, X.-d. (2006). Study on chemical constituents of bamboo grass. *Acta Sci. Natur. Univ. NeiMongol.* 37(2)**,** 175-179.

Lai, C.-Y., Tsai, A.-C., Chen, M.-C., Chang, L.-H., Sun, H.-L., Chang, Y.-L., et al. (2012). Aciculatin induces p53-dependent apoptosis via MDM2 depletion in human cancer cells in vitro and in vivo. *PloS One* 7(8)**,** e42192. doi: 10.1371/journal.pone.0042192.

Neamsuvan, O., Singdam, P., Yingcharoen, K., and Sengnon, N. (2012). A survey of medicinal plants in mangrove and beach forests from sating Phra Peninsula, Songkhla Province, Thailand. *J. Med. Plants. Res.* 6(12)**,** 2421-2437. doi: doi.org/10.5897/JMPR11.1395.

Peter, S.L., and Thomas, B. (2015). Some Medicinal Grasses In Thalappilly Taluk Of Thrissur District, Kerala, India. *Am. J. Biol. Pharm. Res.* 2(3)**,** 135-138.

Pueblos, K.R.S., Bajalla, M., Pacheco, D., Ganot, S., Paig, D., Tapales, R., et al. (Year). "Comparative anthelmintic activity investigation of selected ethno-medicinal weeds", in: *AIP Conference Proceedings*: AIP Publishing), 020027.

Ruizo, E.K.C. (2013). *Phytochemical Screening and In Vitro Antibacterial Activity of Crude Extracts from Andropogon aciculatus retz. (Poaceae).* Bachelor of Science in Biology, University of the Philippines Visayas.

Shahnaj, S., Asha, U., Mim, T., Rumi, N.S.H., Akter, S., Ghose, S.R., et al. (2015). A survey on the ethnomedicinal practices of a folk medicinal practitioner in Manikganj district, Bangladesh *J. Chem. Pharm. Res.* 7(8)**,** 690-696.

Shen, C.-C., Cheng, J.-J., Lay, H.-L., Wu, S.-Y., Ni, C.-L., Teng, C.-M., et al. (2012). Cytotoxic apigenin derivatives from *Chrysopogon aciculatis*. *J. Nat. Prod.* 75(2)**,** 198-201. doi: 10.1021/np2007796.

Shih, K.-S., Wang, J.-H., Wu, Y.-W., Teng, C.-M., Chen, C.-C., and Yang, C.-R. (2012). Aciculatin inhibits granulocyte colony-stimulating factor production by human interleukin 1β-stimulated fibroblast-like synoviocytes. *PloS one* 7(7)**,** e42389. doi: 10.1371/journal.pone.0042389.

Sohel, M., Kawsar, M., Sumon, M., and Sultana, T. (2016). Ethnomedicinal Studies of Lalmohan Thana in Bhola District, Bangladesh. *Altern. Integr. Med.* 5(210)**,** 2. doi: 10.4172/2327-5162.1000210.

Stuart, G. (2016). *Philippine Medicinal Plants* [Online]. Available: <http://www.stuartxchange.org/Amor-seco.html> [Accessed 4th October 2017].

Su, L. (2012). *Method for extracting nudaphantin from Chrysopogon aciculatus*. CN 102827181 A 2012-12-19 China. F.Z. Shenqing.

Tangjang, S., Namsa, N.D., Aran, C., and Litin, A. (2011). An ethnobotanical survey of medicinal plants in the Eastern Himalayan zone of Arunachal Pradesh, India. *J. Ethnopharmacol.* 134(1)**,** 18-25. doi: 10.1016/j.jep.2010.11.053.
